# An Immune-Related Long Non-Coding RNA Signature to Predict the Prognosis of Ewing’s Sarcoma Based on a Machine Learning Iterative Lasso Regression

**DOI:** 10.3389/fcell.2021.651593

**Published:** 2021-05-26

**Authors:** En-hui Ren, Ya-jun Deng, Wen-hua Yuan, Guang-zhi Zhang, Zuo-long Wu, Chun-ying Li, Qi-qi Xie

**Affiliations:** ^1^Breast Disease Diagnosis and Treatment Center, Affiliated Hospital of Qinghai University, Affiliated Cancer Hospital of Qinghai University, Xining, China; ^2^Department of Orthopaedics, Lanzhou University Second Hospital, Lanzhou, China; ^3^The Fourth People’s Hospital of Qinghai Province, Xining, China

**Keywords:** Ewing sarcoma, prognostic analysis, machine learning, immune infiltration, long non-coding RNA

## Abstract

The aim of this study was to construct a new immune-associated long non-coding RNA (lncRNA) signature to predict the prognosis of Ewing sarcoma (ES) and explore its molecular mechanisms. We downloaded transcriptome and clinical prognosis data from the Gene Expression Omnibus (GSE17679, which included 88 ES samples and 18 matched normal skeletal muscle samples), and used it as a training set to identify immune-related lncRNAs with different expression levels in ES. Univariable Cox regression was used to screen immune-related lncRNAs related to ES prognosis, and an immune-related lncRNA signature was constructed based on machine learning iterative lasso regression. An external verification set was used to confirm the predictive ability of the signature. Clinical feature subgroup analysis was used to explore whether the signature was an independent prognostic factor. In addition, CIBERSORT was used to explore immune cell infiltration in the high- and low-risk groups, and to analyze the correlations between the lncRNA signature and immune cell levels. Gene set enrichment and variation analyses were used to explore the possible regulatory mechanisms of the immune-related lncRNAs in ES. We also analyzed the expression of 17 common immunotherapy targets in the high- and low-risk groups to identify any that may be regulated by immune-related lncRNAs. We screened 35 immune-related lncRNAs by univariate Cox regression. Based on this, an immune-related 11-lncRNA signature was generated by machine learning iterative lasso regression. Analysis of the external validation set confirmed its high predictive ability. DPP10 antisense RNA 3 was negatively correlated with resting dendritic cell, neutrophil, and γδ T cell infiltration, and long intergenic non-protein coding RNA 1398 was positively correlated with resting dendritic cells and M2 macrophages. These lncRNAs may affect ES prognosis by regulating GSE17721_CTRL_VS_PAM3CSK4_12H_BMDC_UP, GSE2770_IL4_ACT_VS_ACT_CD4_TCELL_48H_UP, GSE29615_CTRL_VS_DAY3_ LAIV_IFLU_VACCINE_PBMC_UP, complement signaling, interleukin 2-signal transducer and activator of transcription 5 signaling, and protein secretion. The immune-related 11-lncRNA signature may also have regulatory effects on the immunotherapy targets CD40 molecule, CD70 molecule, and CD276 molecule. In conclusion, we constructed a new immune-related 11-lncRNA signature that can stratify the prognoses of patients with ES.

## Introduction

Ewing sarcoma (ES) is one of the most common malignant tumors in children, young adults, and adults (Grünewald et al., 2018). In the past two decades, there has been great progress in ES treatment, through surgery, radiotherapy, and intensive chemotherapy (Pappo and Dirksen, 2018), and patient prognosis has significantly improved. The current 5-year survival rate of patients with local ES is >70%; however, the 5-year survival rate of patients with metastatic or recurrent ES tumors remains at only 20–30% (Burdach and Jürgens, 2002; Iwamoto, 2007). Unfortunately, breakthroughs in the treatment of recurrent and metastatic ES have been difficult to achieve. The precise classification of patients with different prognoses is crucial for precise ES treatment. ES prognosis is closely related to immune factors. For example, CD8+ T cells can kill ES cells by specifically recognizing the ET-derived antigens enhancer of zeste 2 polycomb repressive complex 2 subunit 666 and chondromodulin 319 (Thiel et al., 2011; Blaeschke et al., 2016). Natural killer (NK) cells do not recognize specific tumor antigens to cause an immune response, but exert a direct killing effect on ES cells. Studies have shown that allogeneic transplantation of NK cells has a more pronounced killing effect on tumors than autologous NK cells (Ljunggren and Malmberg, 2007; Verhoeven et al., 2008). In addition, macrophages, mast cells, antigen presenting cells, and dendritic cells are also involved in the molecular mechanisms of ES (Dagher et al., 2002; Lau et al., 2007; Inagaki et al., 2016; Fedorova et al., 2019); however, their specific roles remain unclear. Studies have shown that interleukin (IL)-6, IL-10, and killer cell lectin like receptor K1 regulate the ES tumor microenvironment and are closely related to its prognosis (Hempel et al., 1997; Berghuis et al., 2012; Lissat et al., 2015). Therefore, immune-related prediction signatures may provide accurate guidance for ES treatment.

The discovery of the first long non-coding RNA (lncRNA) (Rinn et al., 2007) sparked an entire field of research regarding their effects and molecular mechanisms in disease. LncRNAs can regulate gene expression through signals, decoys, guides, and scaffolds (Ingolia et al., 2011). Increasing studies have shown that lncRNAs can not only regulate immune responses, but also play important roles in the molecular mechanisms controlling tumors, and are closely related to their prognosis (Peng et al., 2017; Ma et al., 2018; Mowel et al., 2018). According to Marques Howarth et al. (2014), the lncRNA Ewing sarcoma associated transcript 1 (EWSAT1) is a downstream target of EWS RNA binding protein 1 (EWSR1), and the proliferation of ES cells can be inhibited by inhibiting EWSAT1 expression. Immune-associated lncRNAs can be used as prognostic biomarkers for glioblastoma multiforme, breast cancer, and bladder cancer (Zhou et al., 2018; Cao et al., 2020; Zhang Y. et al., 2020). However, due to the lack of research on lncRNAs involved in the molecular mechanisms of ES, an immune-related lncRNA prognosis signature has not been reported. In this study, we have identified lncRNAs strongly related to ES prognosis and used machine learning iterative lasso regression to generate and validate an immune-related 11-lncRNA signature that can predict ES prognosis. We also explored its correlations with immune cell infiltration, to provide accurate and reliable guidance for clinical individualized treatment.

## Materials and Methods

### ES Source Data and Identification of Differentially Expressed Immune-Related lncRNAs

Transcriptome data and corresponding clinical data from the GSE17679 dataset were downloaded from the Gene Expression Omnibus^1^. The dataset includes 88 ES samples and 18 matched healthy skeletal muscle samples, and was used as the training set. The immune scores of the 88 ES samples were calculated using the ESTIMATE algorithm (Yoshihara et al., 2013, https://bioinformatics.mdanderson.org/public-software/estimate/), and they were divided into high and low immune infiltration groups accordingly. The Stromal scores, ESTIMATE scores, and tumor purity levels of the two groups were evaluated. The limma package was used to compare the transcriptome data between the groups to identify immune-related lncRNAs, and differential expression analysis was performed between the ES samples and healthy skeletal muscle samples to identify differentially expressed lncRNAs. The intersection of immune-related lncRNAs and differentially expressed lncRNAs is regarded as immune-related and differentially expressed lncRNAs. Transcriptome and clinical data from 58 cases of ES were downloaded from the International Cancer Genome Consortium database for use as an external validation set.

### Construction of an Optimal Immune-Related lncRNA Predictive Signature

A conditional probability survival graph can describe the survival of patients at different time stages in detail (Latenstein et al., 2020). In this study, 88 patients with ES were used to construct a predictive model, and the conditional survival rate was determined using a conditional probability survival curve. Univariate Cox regression was used to identify lncRNAs associated with ES prognosis. The screening criterion was *p* < 0.05. Lasso regression (Frost and Amos, 2017) is mainly used for the supervised learning of high-dimensional data. Each iteration of the regression produces a gene combination related to prognosis. We conducted 500 lasso regressions on candidate lncRNAs, and considered the lncRNA combination with the largest area under curve (AUC) of the receiver operating characteristic (ROC) as the optimal lncRNA signature (Sveen et al., 2012). We also evaluated the optimal prognosis ability of the lncRNA signature, in terms of overall survival time and lncRNA expression according to risk score.

### Verification of the Optimal Immune-Related lncRNA Predictive Signature

To evaluate the reliability of the immune-related lncRNA signature, we evaluated its prognostic value using the external validation set, and calculated the AUC after 3, 5, and 8 years. We also compared the prognostic value of the immune-related lncRNA signature with established ES prognostic biomarkers (BIK, EGFR, CD44, and LGR5) using the external validation set.

Age, gender, and metastasis are common factors affecting ES. To detect whether the optimal immune-associated lncRNA signature was an independent prognostic factor, Kaplan-Meier survival analysis was used to compare the survival of the high and low-risk groups based on these clinical characteristics. Time-dependent AUC analysis can evaluate the consistency indices of different models based on that of the survival model, and was used to verify the accuracy of the lncRNA signature in predicting ES prognosis (compared with individual clinical characteristics and the lncRNA signature and clinical characteristics combined).

### Analysis of Correlations Between lncRNAs in the Optimal Signature and Immune Cells

CIBERSORT (Newman et al., 2019) is an online tool for immune cell subtype deconvolution based on the principle of linear support vector regression. It can use transcriptome data to evaluate the infiltration of 22 types of immune cells. We used CIBERSORT to analyze immune cell infiltration in the high- and low-risk groups. PCA clustering was performed on the filtered data to detect differences between the groups, and the ggplot2 package was used for visualization. The corrplot, ggplot2, and igraph packages were used in R to visualize the correlations, infiltration differences, and interactions, respectively, between 22 kinds of immune cells. Kaplan-Meier analysis was used to explore relationships between immune cell infiltration and ES prognosis. To explore correlations between the lncRNA signature and prognosis-related immune cell infiltration, Pearson correlation analysis was performed and the ggplot2 package was used for visualization.

### Exploration of Immune Checkpoints and Related Pathways

CD27 molecule, CD40, CD70, TNF receptor superfamily member 14, CD276, V-set domain containing T cell activation inhibitor 1, indoleamine 2,3-dioxygenase 1, programmed cell death 1, CD274 molecule, programmed cell death 1 ligand 2, hepatitis A virus cellular receptor 2, T cell immunoreceptor with Ig and ITIM domains, cytotoxic T-lymphocyte associated protein 4, CD86 molecule, inducible T cell co-stimulator, lymphocyte activating 3, and CD58 molecule are the most common immune checkpoint markers used in tumor research. We explored the expression of these common immune checkpoints in the high- and low-risk groups.

To explore the enrichment of important pathways in the high-risk group, we conducted gene set enrichment analysis (GSEA; Subramanian et al., 2005) and gene set variation analysis (GSVA; Hänzelmann et al., 2013). GSEA was performed in GSEA 4.0.3 using “h.all.v7.1.symbols.gmt” and “c7.all.v7.1.symbols.gmt” as reference gene sets. Nominal *p*-values < 0.05 and false discovery rates < 0.05 were considered significant. GSVA was performed on the “h.all.v7.1 symbols.gmt” gene set using the cluster profiler and gsva packages.

## Results

### Identification of Differentially Expressed Immune-Related lncRNAs

**FIGURE 1:**
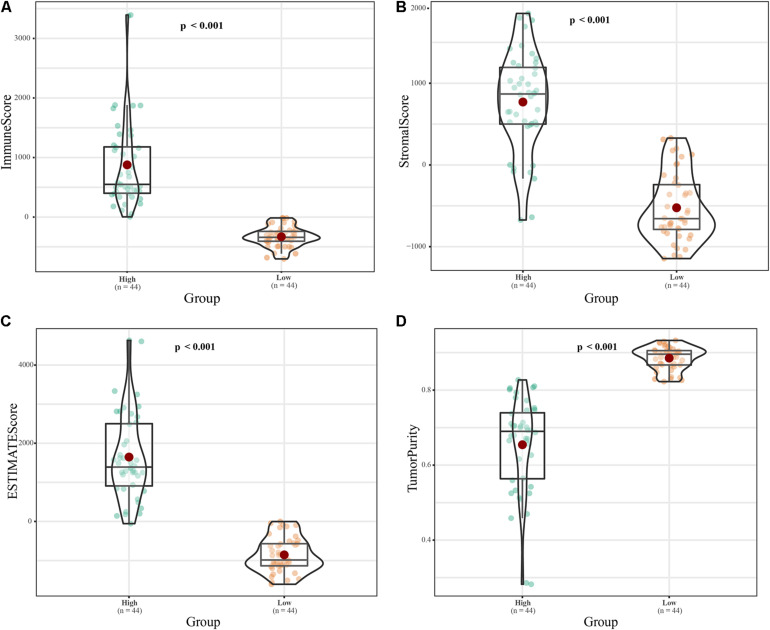
Classification of 88 ES samples based on immune score. **(A)** Box plot of the immune scores of the high- and low-risk groups. **(B)** Box plot of stromal scores. **(C)** Box plot of ESTIMATE scores. **(D)** Box diagram of tumor purity.

The tumor purity of the samples were evaluated using the time-of-life method. According to their immune scores, the 88 ES samples were divided into high and low immune cell infiltration groups (*n* = 44 each; Figure 1A). The ESTIMATE scores (*p* < 0.001) and the Stromal scores (*p* < 0.001) was higher in high immune cell infiltration group, while tumor purity (*p* < 0.001) was lower (Figures 1A–D). Principal component analysis (PCA; Supplementary Figures 1A–D) revealed dramatic differences between the two groups. We obtained 262 immune-related lncRNAs by differential expression analysis of the high and low immune infiltration groups, and 884 differentially expressed lncRNAs in the 88 ES samples compared to the 18 matched healthy skeletal muscle samples. The intersection of these groups of lncRNAs yielded 171 immune-related and differentially expressed lncRNAs (Supplementary Figure 1E).

### Construction of an Immune-Related lncRNA Prognostic Signature

**FIGURE 2:**
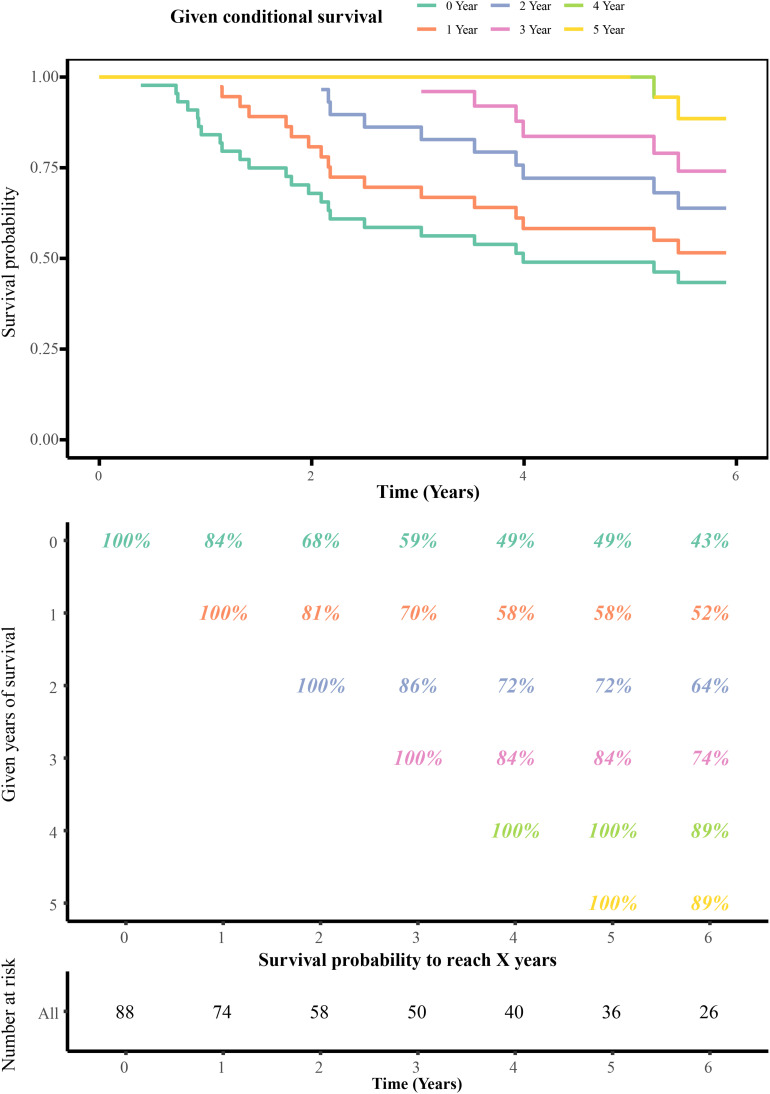
Conditional survival estimates over time. Each column represents a survival period, and each row represents the percentage to reach a certain survival time from that point (in years).

**FIGURE 3:**
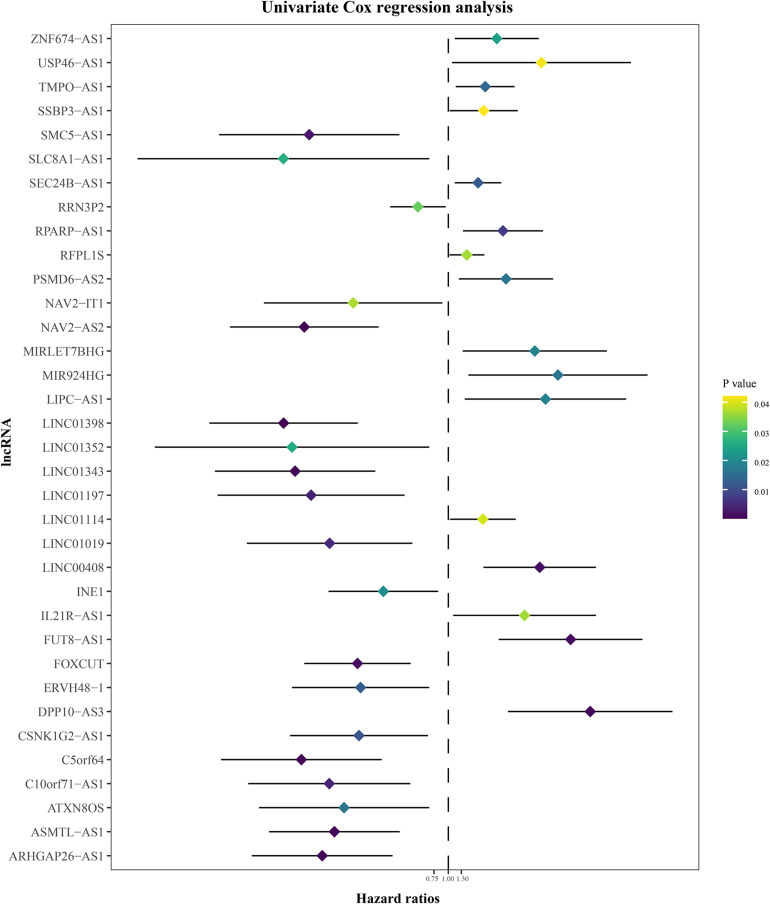
Univariate Cox regression analysis of immune-related differentially expressed lncRNAs.

**FIGURE 4:**
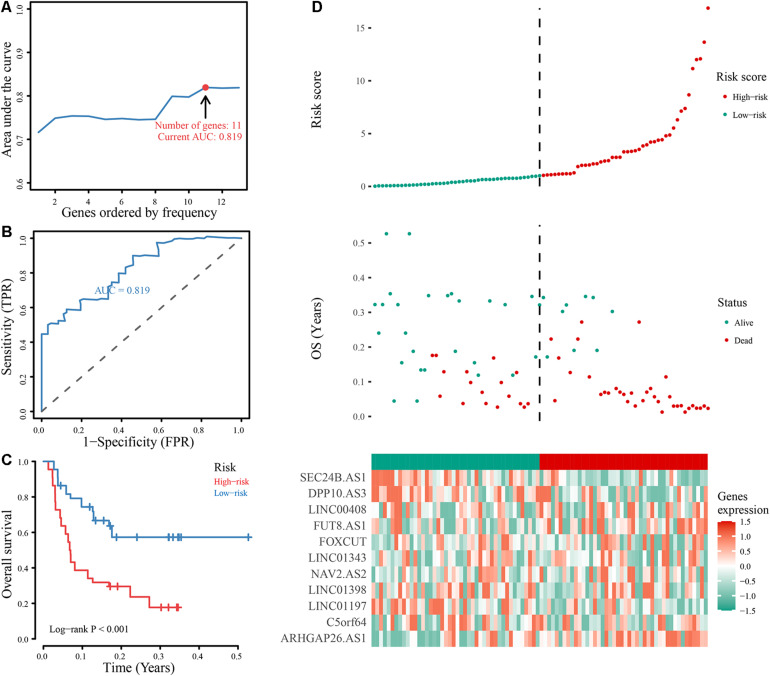
Construction and evaluation of the optimal immune-related lncRNA signature. **(A)** Line chart of the AUC of different immune-related lncRNA signature models. **(B)** ROC curve of the immune-related 11-lncRNA signature. **(C)** Kaplan-Meier analysis of the signature. Survival was compared using the log-rank test. **(D)** Evaluation of the 11-lncRNA signature based on risk factors in the high- and low-risk groups, the RFS, and gene expression in the signature.

The annual conditional survival probability increased with the overall survival time (Figure 2). From a 49% chance of survival immediately post-resection, the probability of 5-year survival 1, 2, 3, and 4 years after resection increased by 58, 72, 84, and 100%, respectively. The probability of surviving the next year decreased from 84% to 81% after 1 year, and then increased to 84 and 89% at 3 and 5 years, respectively. The univariate Cox regression model identified 35 correlations between immune-related differentially expressed lncRNAs and patient prognosis (Figure 3). Prognosis-related lncRNAs were cross-validated via 500 lasso regressions to reveal an optimal immune-related lncRNA prognostic model consisting of 11 lncRNAs (Figure 4A). Receiver operating characteristic (ROC) analysis was used to further evaluate the predictive performance of the immune-related lncRNA prognostic signature. The results show that it has good performance in predicting ES prognosis (AUC = 0.819; Figure 4B). When the patients were divided into high- and low-risk groups according to their risk scores, Kaplan-Meier survival analysis showed that ES prognosis was significantly worse in the high-risk group (log-rank *p* < 0.001, Figure 4C). Both the risk scores and the number of deaths in the high-risk group were significantly higher than those of the low-risk group (Figure 4D). Of the 11 immune-related lncRNAs, ARHGAP26 antisense RNA 1 (ARHGAP26-AS1), FUT8 antisense RNA 1 (FUT8-AS1), FOXC1 upstream transcript (FOXCUT), and chromosome 5 putative open reading frame 64 (C5orf64) were highly expressed in the high-risk group, while NAV2 antisense RNA 2 (NAV2-AS2), long intergenic non-protein coding RNA (LINC)00408, SEC24B antisense RNA 1 (SEC24B-AS1), LINC01343, LINC01398, LINC01197, and DPP10 antisense RNA 3 (DPP10-AS3) were lowly expressed in the high-risk group (Figure 4D).

### Verification of the Optimal Immune-Related lncRNA Signature

**FIGURE 5:**
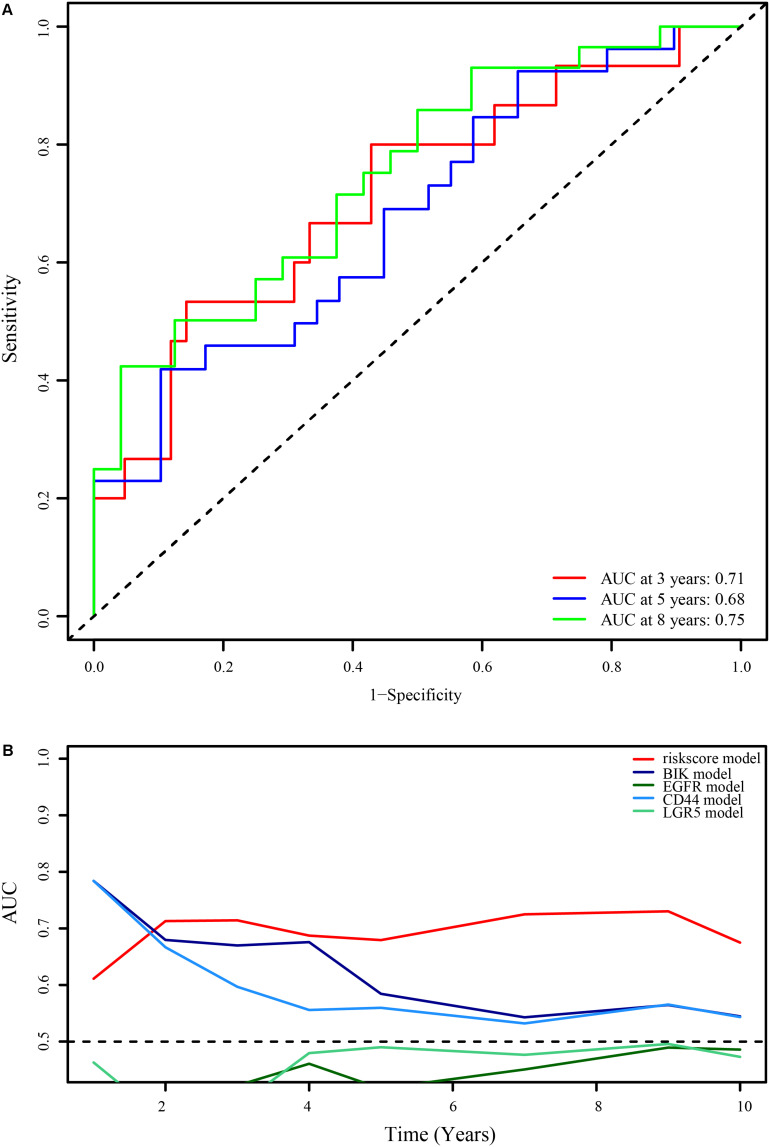
Verification of the optimal immune-related 11-lncRNA signature. **(A)** 3-, 5-, and 8-year ROC curves of the 11-lncRNA signature in the external verification set. **(B)** Comparison of the 11-lncRNA signature and common prognostic biomarkers of ES.

To verify the reliability of the optimal immune lncRNA signature, we evaluated its predictive value in 58 ES samples in the external validation set through ROC analysis. The immune-related lncRNA signature had obvious prognostic value after 3 (AUC = 0.71), 5 (AUC = 0.68), and 8 years (AUC = 0.75; Figure 5A). Compared with prognostic biomarkers such as BCL2 interacting killer (BIK), epidermal growth factor receptor (EGFR), CD44 molecule (Indian blood group) (CD44), and leucine rich repeat containing G protein-coupled receptor 5 (LGR5), the lncRNA signature had better prognostic value (Figure 5B).

### Evaluation of the Immune-Related lncRNA Signature as an Independent ES Prognostic Factor and Its Prediction Accuracy

**FIGURE 6:**
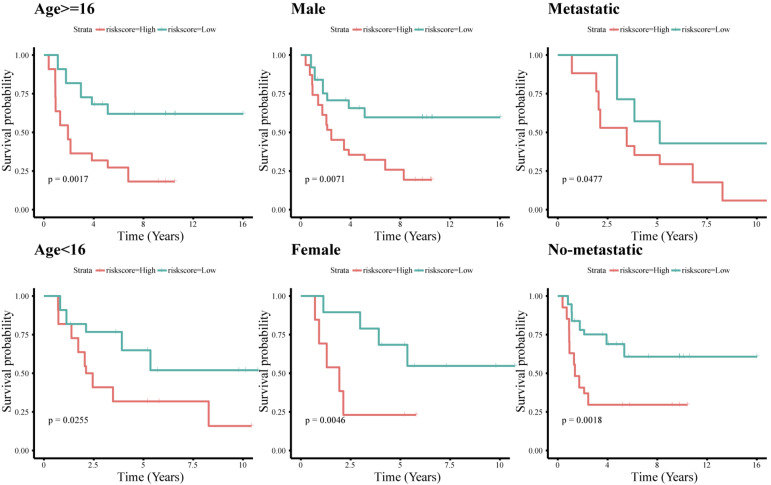
Survival analysis of the immune-related 11-lncRNA signature with different clinical characteristics.

**FIGURE 7:**
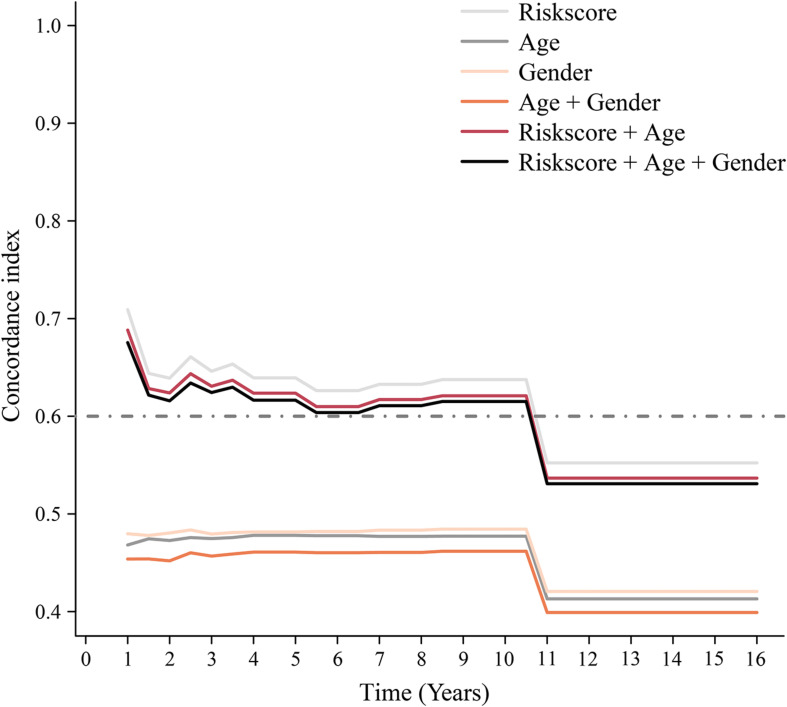
Time-dependent ROC curve. Concordance index (cindex) shows measure of concordance of predictor with survival of patients. The minimum threshold for cindex to be considered effective is 0.6.

To assess whether the immune-related lncRNA signature acts is a prognostic factor independent of clinical characteristics (age, sex, and metastasis), Kaplan-Meier survival analysis was performed in different subgroups of the high- and low-risk groups. Prognosis was poor in all subgroups of the high-risk group (*p* < 0.05, Figure 6), suggesting that the signature is independent of age, sex, and metastasis. The lncRNA signature was more accurate in predicting ES prognosis than age, sex, age + sex, and age + sex + lncRNA signature models (Figure 7).

### Correlations Between the Immune-Related lncRNA Prognostic Signature and Immune Cell Subtype Infiltration

**FIGURE 8:**
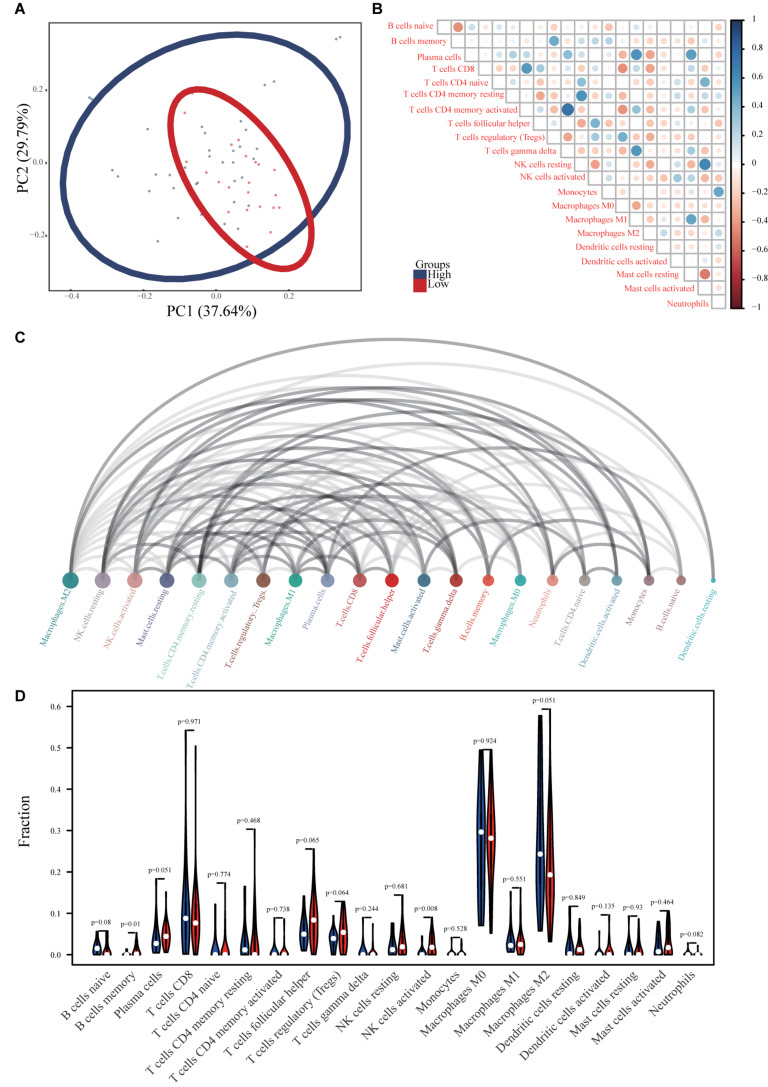
Immune cell infiltration in ES. **(A)** PCA analysis of immune cell infiltration in ES samples and healthy skeletal muscle samples. **(B)** Correlation heat maps of 22 immune cell types. The size of each colored circle represents the related *p*-value; the color represents the strength of the correlation (blue and red indicate positive and negative correlations, respectively, with darker colors indicating stronger correlations). **(C)** Interaction network between 22 immune cell types. The circle size represents the strength of the interaction. **(D)** Violin chart showing differences in the infiltration of 22 immune cell types in the ES and healthy skeletal muscle groups.

**FIGURE 9:**
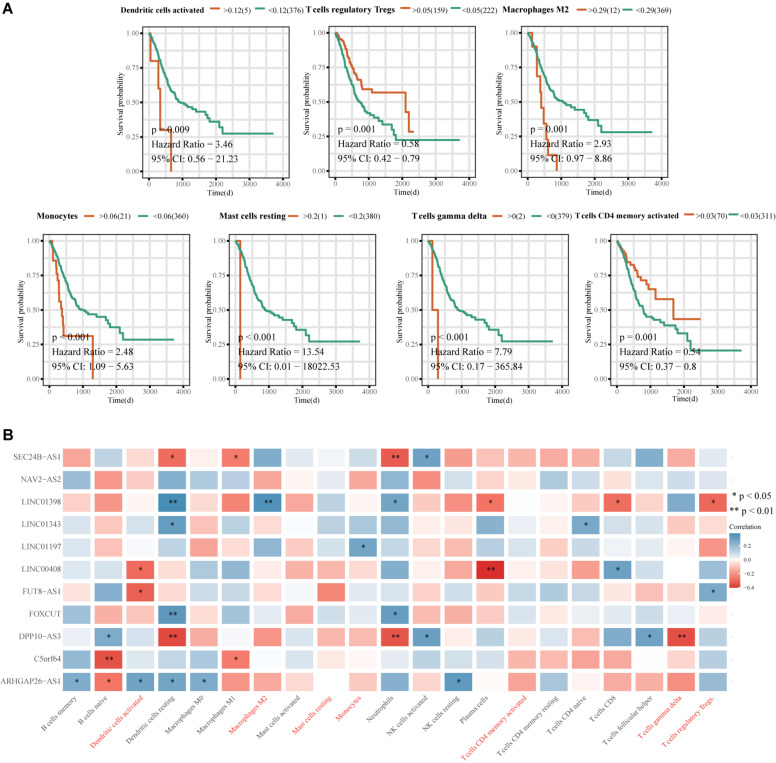
Correlation analysis between the immune-related 11-lncRNA signature and prognosis-related immune cells. **(A)** Kaplan-Meier survival analysis of immune cell infiltration and ES prognosis. **(B)** Heat map of correlations between the 11-lncRNA signature and prognostic immune cells. The ordinate is the gene name, the abscissa is the immune cell type, and the color represents the correlation coefficient; ^∗^*p* < 0.05, ^∗∗^*p* < 0.01.

Principal component analysis of the high- and low-risk groups revealed differences in immune cell infiltration (Figure 8A). Correlation analysis showed that plasma cells were positively correlated with M1 macrophages and resting mast cells, but negatively correlated with M2 macrophages. activated T cells CD4 memory were positively correlated with γδ T cells and negatively correlated with M0 macrophages (Figure 8B). M2 macrophages, resting NK cells, and activated NK cells had the strongest interactions with other immune cells, while monocytes, naïve B cells, and resting dendritic cells had the weakest interactions (Figure 8C). Memory B cells and activated NK cells showed higher infiltration in the high-risk group compared to the low-risk group (Figure 8D). Infiltration of regulatory T cells (Tregs; *p* = 0.001) and activated CD4 memory T cells (*p* = 0.001) indicated good ES prognosis, while infiltration by activated dendritic cells (*p* = 0.009), M2 macrophages (*p* = 0.001), monocytes (*p* < 0.001), resting mast cells (*p* < 0.001), and γδ T cells (*p* < 0.001) indicated poor prognosis (Figure 9A). DPP10-AS3 was positively correlated with resting dendritic cell, neutrophil, and γδ T cell infiltration, while LINC01398 was negatively correlated with resting dendritic cell and M2 macrophage infiltration (Figure 9B).

### Signature-Related Pathways and Immune Checkpoint Markers

**FIGURE 10:**
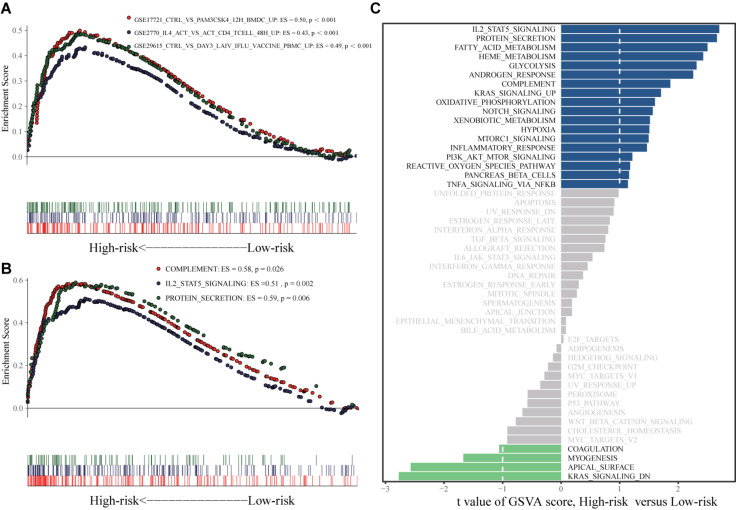
GSEA and GSVA analyses. **(A)** GSEA analysis based on h.all.v7.1.symbols.gmt. **(B)** GSEA analysis based on c7.all.v7.1.symbols.gmt. **(C)** GSVA analysis.

The results of gene set enrichment analysis (GSEA) are shown in Figures 10A,B. Pathways enriched in the high-risk group included GSE17721_CTRL_VS_PAM3CSK4_12H_BMDC_UP, GSE2770_IL4_ACT_VS_ACT_CD4_TCELL_48H_UP, GSE29615_CTRL_VS_DAY3_LAIV_STAT_VACCEMENT, REMARK_MARKINHALL_COM_PLTION, REMARK_COM_PL_COM, REMARK_COMP_UP, and REMARK_COMP_PROG. Gene set variation analysis (GSVA) revealed activation of IL2-signal transducer and activator of transcription 5, protein secretion, complement, and phosphoinositide 3-kinase (PI3K)-Akt-mammalian target of rapamycin signaling in the high-risk group (Figure 10C).

**FIGURE 11:**
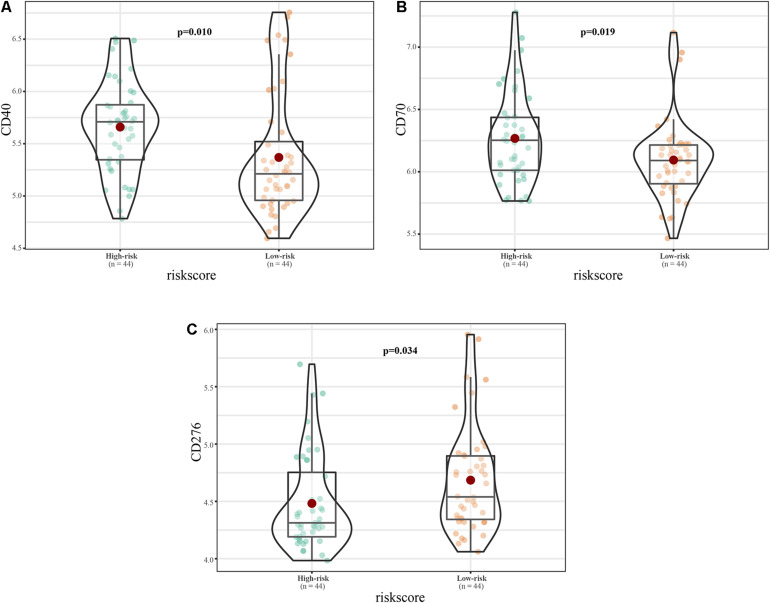
Immune checkpoint expression in the high and low risk groups. **(A–C)** Expression of **(A)** CD40, **(B)** CD70, and **(C)** CD276.

Among common immune checkpoint markers, the levels of CD40 molecule (CD40; *p* = 0.01) and CD70 molecule (CD70; *p* = 0.019) were higher in the high-risk group, while CD276 molecule (CD276; *p* = 0.019) was higher in the low-risk group (Figure 11).

## Discussion

Increasing studies have shown that lncRNAs play important roles in the occurrence and development of various tumors. LncRNAs are not only involved in tumor regulatory mechanisms, but their levels are also closely related to patient prognosis. For example, the lncRNA CBR3 antisense RNA 1 can not only promote the occurrence of osteosarcoma by regulating the proliferation, migration, invasion, and apoptosis of osteosarcoma cells, but is also an independent prognostic factor of the disease (Zhang et al., 2018). This study aimed to identify an optimal immune-related lncRNA signature to predict the prognosis of ES. After screening for prognosis-related lncRNAs, the 11-lncRNA signature was constructed using a machine learning-iterative lasso regression model. Compared with the traditional stepwise regression method for constructing prognostic signatures, this method is based on the penalized lasso regression method, and combines lncRNAs with strong prognostic correlations to obtain optimal lncRNA signatures (Friedman et al., 2010; Goeman, 2010). This method not only considers the prognostic information of each lncRNA, but also removes redundant prognostic information, maximizing the prognostic value of the lncRNA signature. We also used bioinformatic methods to explore relationships between the lncRNA signature and prognosis-related immune cells, and explored the potential regulatory mechanisms involved, providing new research avenues in the study of immune-related lncRNAs in ES.

We identified 11 differentially expressed immune-related lncRNAs: ARHGAP26-AS1, FUT8-AS1, FOXCUT, C5orf64, NAV2-AS2, LINC00408, SEC24B-AS1, LINC01343, LINC01398, LINC01197, and DPP10-AS3. NAV2-AS2 and SEC24B-AS1 are prognostic biomarkers for lung adenocarcinoma (He and Zuo, 2019) and non-small cell lung cancer (Yang et al., 2020), respectively. Zhang X. et al. (2020) showed that FOXCUT promotes the metastasis and proliferation of colorectal cancer by activating the forkhead box C1/PI3K/Akt pathway. In addition, FOXCUT plays important roles in the molecular mechanisms of breast cancer, nasopharyngeal carcinoma, gastric adenocarcinoma, and esophageal squamous cell carcinoma, and can be used as a prognostic biomarker of esophageal squamous cell carcinoma (Pan et al., 2014; Liu et al., 2015; Xu et al., 2017; Zhao and Shen, 2019). The relationships between the 11 lncRNAs and ES are currently unclear, and biological studies will be required to explore their roles in its molecular mechanisms. Our results demonstrate that the immune-related 11-lncRNA signature has a higher prognostic value than other known prognostic biomarkers and is not affected by clinical characteristics. Therefore, the signature has strong prognostic evaluation value. However, large-scale experimental verification will be required before it can be accurately and reliably used in the clinic.

To evaluate immune cell infiltration in ES, we applied the deconvolution method to analyze ES expression data and found that memory B cells and activated NK cells had higher infiltration in the high-risk group than in the low-risk group, and that Tregs, activated CD4 memory T cells, activated dendritic cells, M2 macrophages, monocytes, resting mast cells, and γδ T cells were significantly related to ES prognosis. The killing effects of NK cells on ES cells have been experimentally verified (Verhoeven et al., 2008). Osteosarcoma displays mast cell infiltration (Inagaki et al., 2016); however, their infiltration of ES is currently unclear. The infiltration of tumor-associated macrophages and mast cells indicates poor ES prognosis (Pollard, 2004; Fujiwara et al., 2011), consistent with our results. In immunodeficient mice, γδ T cells can mediate the cytotoxicity of antibody-dependent ES cells with high expression of GD2 (Fisher et al., 2016). Guo et al. (2008) reported that dendritic cells have strong inhibitory effects on the proliferation of subcutaneous ES cells in mice. The roles of memory B cells, activated CD4 memory T cells, and Tregs in the occurrence and development of ES have not been reported. We also found that DPP10-AS3 was negatively correlated with resting dendritic cell, neutrophil, and γδ T cell infiltration, and LINC01398 was positively correlated with resting dendritic cell and M2 macrophage infiltration. However, the relationships between these lncRNAs and these prognostic-related immune cell types remain unclear, and will require further biological experimentation.

In GSEA and GSVA, GSE17721_CTRL_VS_PAM3CSK4_12H _BMDC_UP, GSE2770_IL4_ACT_VS_ACT_CD4_TCELL_48H_ UP, GSE29615_CTRL_VS_DAY3_LAIV_IFLU_VACCINE_ PBMC_UPLING, HSTATALLMARK_COMPLEMENT2_HALL MARK_MARK_UP_HALL_MARK_COMPLTION, and HSTAT ALL_COMPLTION_HALLMARK_COMPLTION_HALLMARK _COMPLTION were significantly enriched in the high risk group compared to the low-risk group. The complement system plays an important role in ES pathogenesis. For example, complement C5 is activated in ES and is positively correlated with better prognosis (Savola et al., 2011). In addition, a decrease in extracellular matrix protein secretion is related to loss of ES cell invasion ability (Javelaud et al., 2002). Therefore, the lncRNAs included in the signature may affect ES prognosis in part by regulating the complement system and the secretion of extracellular matrix proteins. The expression of the EWSR1-WT1, a fusion protein containing sections of EWSR1 and WT1 transcription factor, in proliferative small round cell tumors can induce the expression of IL-2 and IL-15, which are related to the proliferation of these tumor cells (Wong et al., 2002). However, the role of the IL-2-STAT5 signaling pathway in the pathogenesis of ES has not yet been reported. The specific role of protein secretion in ES pathogenesis also remains unclear, and further research is needed. Further study of these pathways will elucidate the immune regulatory mechanisms of ES.

Among common immune checkpoint markers, the levels of CD40, CD70, and CD276 differed between the high- and low-risk groups. Lollini et al. (1998) analyzed the expression of CD40 in 12 human osteosarcoma cell lines, six ES lines, and five rhabdomyosarcoma lines by flow cytometry. CD40 was highly expressed in osteosarcoma and ES, and was closely related to ES prognosis. CD70 is a therapeutic target for osteosarcoma (Pahl et al., 2015); however, its role in ES pathogenesis is currently unclear. CD276 is an immunotherapy target for peritoneal cancer, glioma, and central nervous tumors (Picarda et al., 2016), and its role in ES is also unclear. Our results show that the 11-lncRNA signature is closely related to these therapeutic targets. However, the specific regulatory relationships between the signature and CD40, CD70, and CD276 will require biological experimentation.

In summary, this study reports the first immune-related lncRNA signature related to ES prognosis. The gene signature is closely related to the infiltration of a variety of immune cell types, and reveals pathways and immune checkpoints that may be regulated by the 11 lncRNAs comprising it.

## Data Availability Statement

The datasets presented in this study can be found in online repositories. The names of the repository/repositories and accession number(s) can be found in the article/Supplementary Material.

## Author Contributions

E-HR, Y-JD, and W-HY: conception and design, data analysis, and manuscript writing and revision. E-HR, Z-LL, and G-ZZ: data sorting and data checking. Q-QX and C-YL: data acquisition, research design, and picture drawing. All authors read and approved the final manuscript.

## Conflict of Interest

The authors declare that the research was conducted in the absence of any commercial or financial relationships that could be construed as a potential conflict of interest.

## Footnotes

1 https://www.ncbi.nlm.nih.gov/geo/

## Abbreviations

EWSAT1: Ewing sarcoma associated transcript 1
EWSR1: EWS RNA binding protein 1
PCA: principal component analysis
ROC: receiver operating characteristic
ARHGAP26-AS1: ARHGAP26 antisense RNA 1
FUT8-AS1: FUT8 antisense RNA 1
FOXCUT: FOXC1 upstream transcript
C5orf64: chromosome 5 putative open reading frame 64
NAV2-AS2: NAV2 antisense RNA 2
LINC: long intergenic non-protein coding RNA
SEC24B-AS1: SEC24B antisense RNA 1
DPP10-AS3: DPP10 antisense RNA 3.
