# Supplementary material for: An Immune-Related Long Non-Coding RNA Signature to Predict the Prognosis of Ewing’s Sarcoma Based on a Machine Learning Iterative Lasso Regression
Source: Front Cell Dev Biol. 2021 May 26;9:651593. doi: 10.3389/fcell.2021.651593 (PMC8187926; doi:10.3389/fcell.2021.651593)
Supplement: Supplementary file 1 [file Data_Sheet_1.docx]

Supplementary Material

## Supplementary Figures
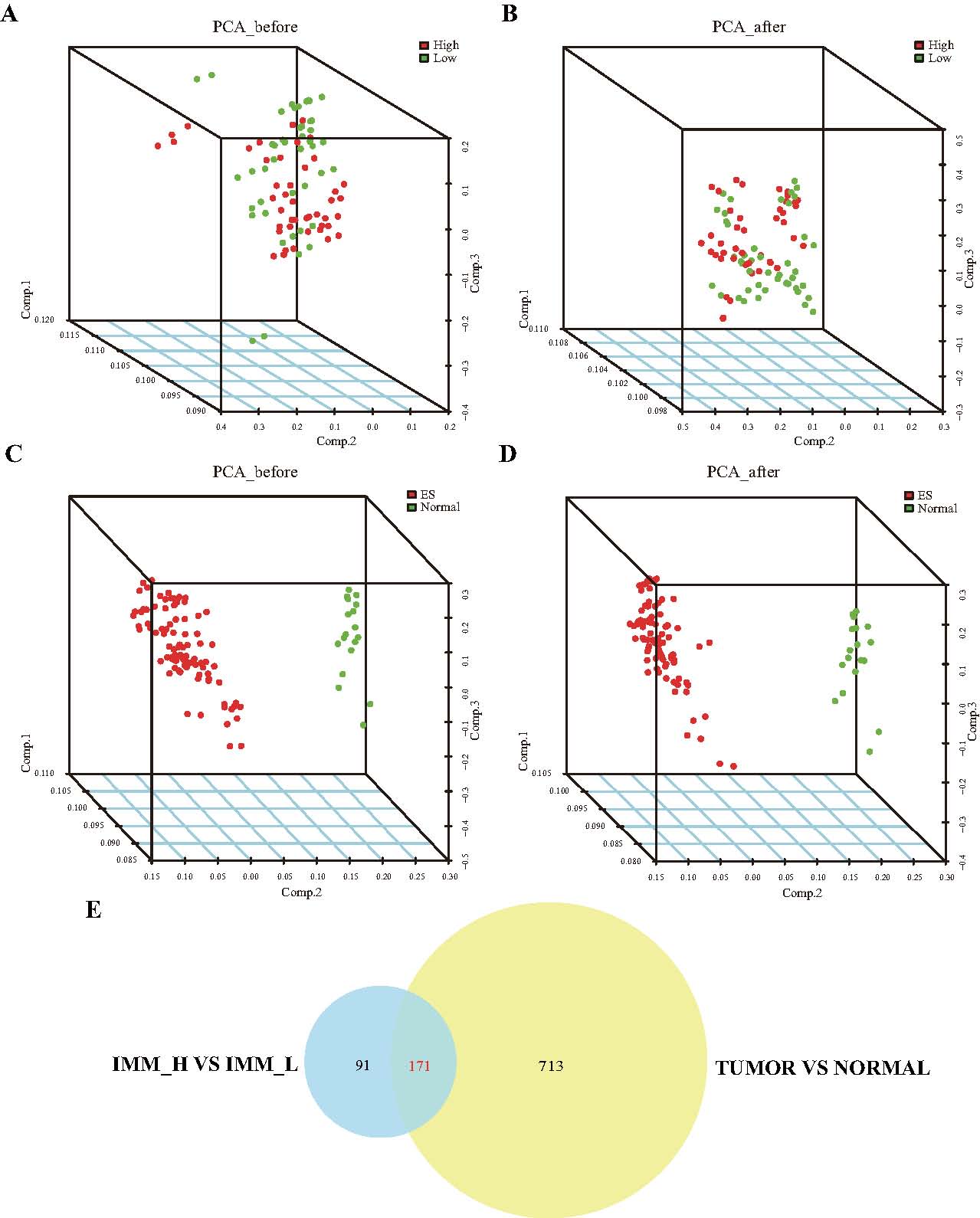


**Supplementary** **Figure 1.** Processing of original data and obtaining of immune lncrna
